# Interference Torque of a Gas-Dynamic Bearing Gyroscope Subject to a Uniform Change of the Specific Force and the Carrier Angular Velocity

**DOI:** 10.3390/s20236852

**Published:** 2020-11-30

**Authors:** Yan Li, Desheng Zhang, Fuhai Duan

**Affiliations:** 1 Research Center of Fluid Machinery Engineering and Technology, Jiangsu University, Zhenjiang 212013, China; li_yan@ujs.edu.cn; 2 School of Mechanical Engineering, Dalian University of Technology, Dalian 116023, China; duanfh@dlut.edu.cn

**Keywords:** gas-dynamic bearing gyroscope, interference torque, five-degrees-of-freedom model

## Abstract

The work is devoted to an analysis of interference torque of a gas-dynamic bearing gyroscope, while a condition with uniformly changed specific force and carrier angular velocity are taken into account. A five-degrees-of-freedom (5-DOF) model is established considering the translation and tilt of the rotor, which solves dynamic rotor equations and the Reynolds equation simultaneously. The model makes it possible to obtain the rotor trajectory under time-transient specific force and carrier angular velocity. The interference torque of the gyroscope is analyzed based on the rotor trajectory. Results indicate that the gas-dynamic bearings show a significant hysteresis effect with a perturbation of bearing force or bearing moment, which indicates the necessity of transient research. Interference torque is large when the carrier angular velocity starts to change or stops to change, and when the specific force stops to change. When the specific force change rate is less than 8.4 km/s^3^ with no change of the carrier angular velocity, the condition could be simplified as a steady state, which is consistent with the previous study.

## 1. Introduction

Gas-dynamic bearing gyroscopes have gained wide acceptance with high precision. Their performance determines the precision of the inertial navigation system to a large extent [1,2,3]. They have been designed with gas-dynamic bearings to support the rotor. As gases are less viscous than liquid, and the environmental temperature has only a little impact on it, the rotational speed of gas-dynamic bearing gyroscopes could reach 10,000~100,000 rotations per minute (rpm) [4]. Moreover, the high speed could greatly reduce the gyro error torque without increasing weight [5,6]. However, the flexibility of gas-dynamic bearings may lead to the rotor-eccentric motion, with the action of the specific force (the difference between acceleration vector and gravity acceleration vector) and the carrier angular velocity. Moreover, an error will occur as a result of the eccentricity [7]. It is challenging to reveal the mechanism of error transfer because of the nonlinear elasticity of the gas-dynamic bearings and the complex five-degrees-of-freedom model (5-DOF) motion of the rotor. The key to correct the error is to predict the interference torque through rotor dynamics research, based on the theory described in the previous studies [7,8].

The dynamic characteristics of a rotor supported by gas-dynamic bearings have been investigated by many scholars. As the gyroscope outputs in real time, it is an effective method to solve the transient Reynolds equation and rotor dynamic equation simultaneously, which can accurately track the rotor trajectory [9]. Meybodi et al. [10] analyzed the effect of mass unbalance on the characteristics of a gas-dynamic four-lobe bearing, with the Runge–Kutta method to track the rotor. Chen [11] applied the Q-Z decomposition method to study the vibration of the stepped shaft supported by gas bearings, dividing the stepped shaft into wheel disc and shaft segment. Bonello et al. [12,13,14] solved the Reynolds equation in the frequency domain, which is applied in the simulation of gas-dynamic foil bearing and rotor dynamics modeling of aero engines. Kim [15] solved the trajectory of the rotor with mass eccentricity and analyzed the condition for stability with the trajectory. Pronobis [16] compared the results obtained by the transient method and the linearized perturbation methods and applied the two methods to calculate the stability limits of a gas foil bearing. Du et al. [17] investigated the nonlinear whirl motion of a rotor supported by a gas-dynamic bearing, with the trajectories and phase portrait being obtained. Wang et al. [18] developed a method incorporating the differential transformation method with the finite difference method to study the coupling characteristics of rotor deformation and gas film pressure. Nielsen et al. [19,20] investigated the transient behaviors of a gas-dynamic foil bearing, including the harmonic vibrations with large journal unbalance. Liu et al. [21] adopted the two-dimensional narrow theory to the model of herringbone grooved journal gas bearings to analyze its stability. Zhang et al. [22] investigated the effects of temperature on the nonlinear dynamic behavior of a gas journal bearing for microengine. Wang [23] analyzed the dynamic response of a three-multilobe air bearing system for different rotor mass and bearing numbers using the differential transformation method and the finite difference method. Hassini [24] developed a novel method to lead a transient nonlinear calculation with linearized dynamic coefficients at different eccentricities, which has a certain improvement in the calculation speed. However, Bouzidane et al. [25] compared the linearized method with the method of solving the transient Reynolds equation and found that the accuracy of the linearized method is obviously lower under the condition of large amplitude. Zhang et al. [26] developed a method to track the rotor by predicting the rotor position in the next time step with states in the previous two steps, which improves the calculation speed too, but the effect of prediction is poor in the position of high nonlinearity, resulting to a divergence of the calculation. Bailey et al. [27] analyzed the dynamic rotor behavior with Navier slip boundary conditions and calculated the minimum clearance for various designs.

Liu [28] studied the interference torque of a gas-floated gyroscope with surface roughness considered. He also assumed several manufacturing errors and analyzed the correlation between manufacturing errors and interference torque. Liang et al. [29] investigated the effects of film thickness, surface roughness, and slit width on the vortex torque of a gas-floated gyroscope. Li et al. [7,30] established a static error model of a three-floated gyroscope with a rotor supported on gas-dynamic bearings and calculated the transient interference torque under the assumption that the specific force changes suddenly. However, the change of carrier angular velocity is ignored, which could lead to a dynamic error. In addition, many changing conditions cannot be simplified as sudden changes, and it is more general to decompose the changes into uniform changes.

In this paper, a model is established considering the 5-DOF motion, including translation and tilt of the rotor. Rotor dynamic equations and the Reynolds equation are solved simultaneously to track the rotor trajectory. Hysteresis effects of the gas-dynamic bearings are studied, which shows the necessity to investigate the transient behavior with a nonlinear model. Transient interference torque curves are obtained considering a uniform change of the specific force and the carrier angular velocity.

## 2. Mechanical Model

**Figure 1:**
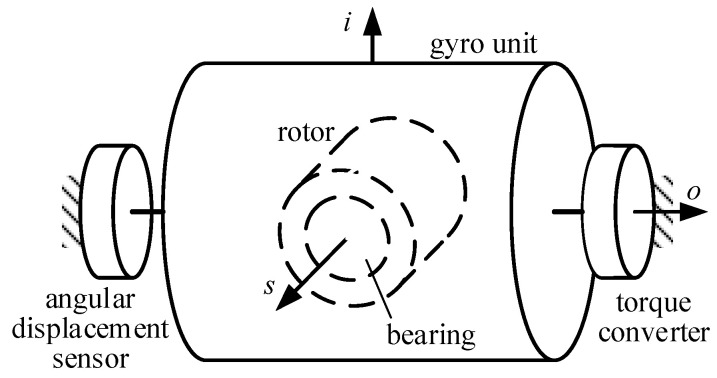
Schematic of a gas-dynamic bearing gyroscope.

**Figure 2:**
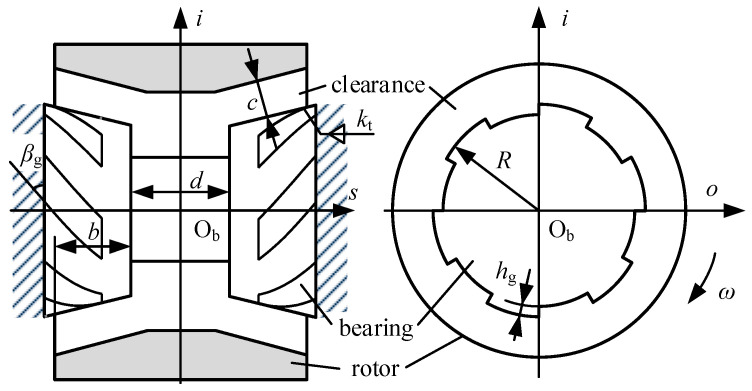
Schematic structure of gas-dynamic bearings.

**Figure 3:**
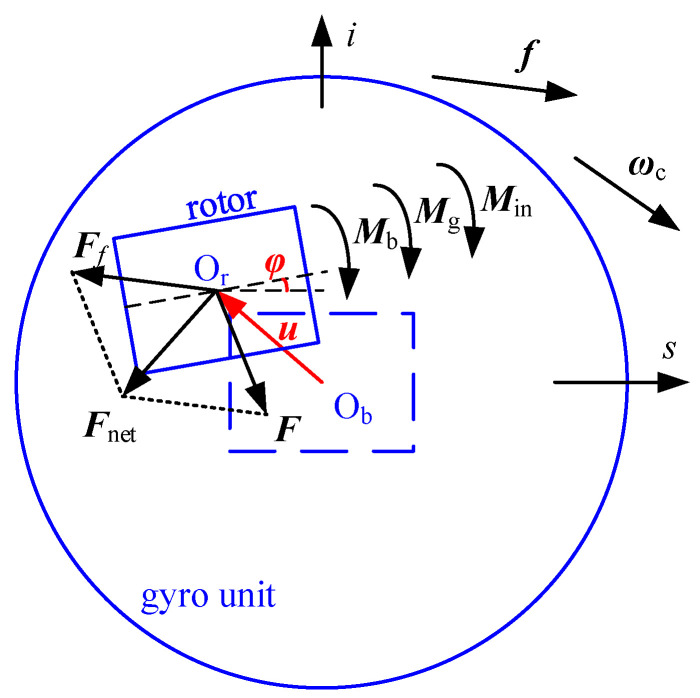
Forces and moments acting on the rotor.

The configuration of the gas-dynamic bearing gyroscope is shown in Figure 1. An angular displacement sensor, a torque converter and the coordinate system O_b_*ios* are fixed on the carrier. The gyro unit could only rotate around the *o*-axis with the limit of the *o*-axis bearing. A pair of conical gas-dynamic bearings shown in Figure 2 are mounted oppositely inside the gyro unit. The rotor rotates at high speed around the *s*-axis and is supported by the gas film in the clearance between the rotor and the bearings. When the carrier rotates around the *i*-axis, the rotor generates a gyroscopic moment around the *o*-axis, which turns the gyro unit around the *o*-axis and influence the current in the angular displacement sensor and the torque converter. The current is detected to estimate the angular velocity of the carrier around the *i*-axis. Under the influence of the carrier transient motion, as shown in Figure 3, the specific force ***f*** causes an apparent gravity ***F****_f_* = −*m**f*** in the non-inertial reference frame O_b_ on the rotor, whose mass is *m*. The carrier angular velocity ***ω***_c_ causes a gyroscopic moment ***M***_g_ on the rotor, and the angular acceleration of the rotor causes an inertial moment ***M***_in_ on the rotor. It is assumed that the rotor and the bearing are both rigid. The 5-DOF eccentric motion of the rotor includes the translation ***u*** = (*u_i_*, *u_o_*, *u_s_*) and the tilting motion *φ_i_*, *φ_o_*, which are the components of rotation ***φ*** = (*φ_i_*, *φ_o_*, *φ_s_*). The 5-DOF eccentric motion could change the clearance and produce bearing force ***F*** and bearing moment ***M***_b_. It is determined by the combined action of the bearing force, the apparent gravity, the bearing moment, the gyroscopic moment, and the inertial moment. As a result, an interference torque ***M*** is produced by the 5-DOF eccentric motion, and its *o*-component ***M****_o_* is expressed by

(1) $$M_{o}=-M_{bo}-u\times F\cdot e_{o}-H_{r}\omega_{ci}$$

where ***e****_o_* is a unit vector in *o*-direction, ***M***_b*o*_ is the *o*-component of ***M***_b_ and *ω*_c*i*_ is the *i*-component of *ω*_c_.

The schematic structure of a gas-dynamic bearing is shown in Figure 2. The rotor is designed to be outside the bearings to get a larger polar moment of inertia, thereby increasing the sensitivity of the gyroscope. The geometry of the bearings is characterized by the bottom radius *R*, the taper *k*_t_ and the width *b*. Grooves are carved on the surfaces in a spiral manner, characterized by groove depth *h_g_* and groove angle *β_g_*. The clearance between the bearings and the rotor is *c*, the spacing between the bearings is *d*, and the angular velocity of the rotor is *ω*.

## 3. Mathematical Model

### 3.1. Governing Equations

The translation and tilting motion of the rotor in the reference frame O_b_*ios* are governed by the following dynamic equations separately

(2) $$mf-F+m\frac{\partial^{2}u}{\partial t^{2}}=0$$

(3) $$J\left(\frac{d^{2}\varphi }{dt^{2}}+\frac{d\omega_{c}}{dt}\right)=M_{b}+J\omega \times \left(\frac{d\varphi }{dt}+\omega_{c}\right)$$

where ***J*** = diag(*J*_d_, *J*_d_, *J*_p_) is the matrix for the moment of inertia, *J*_d_ is the moment of inertia of the rotor around the *i*-axis or *o*-axis, *J*_p_ is the moment of inertia of the rotor around the *s*-axis. As the rotation around *s*-axis *φ_s_* is controlled by the current in the rotor winding, the 5-DOF motion of interest does not include *φ_s_*. However, *φ_s_* is listed as a component of ***φ*** only for the convenience of calculation.

**Figure 4:**
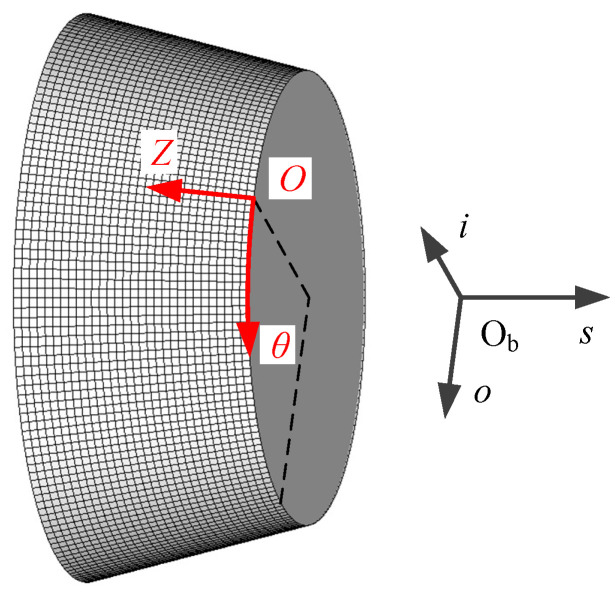
Coordinate systems and grid system on the bearing surface.

The bearing force and the bearing moment produced by the gas film are calculated from the integration of the pressure in the gas film, which is governed by the Reynolds equation for compressible gas. The gas film is assumed to be isothermal as a result of the temperature control system. The Knudsen number is around 0.03 calculated with the clearance *c* = 2 μm, the ambient pressure *P*_a_ = 101.325 kPa and the ambient temperature *T*_a_ = 293 K. Hence, the effect of gas rarefaction needs to be considered, and the F-K model is adopted which is applicable for an arbitrary Knudsen number as a result of the rotor movement. Considering all mentioned above, the following modified Reynolds Equation (4) for the conical gas film is established in the coordinate system O*θZ* shown in Figure 4.

(4) $$\frac{\partial }{\partial Z}\left(pQh^{3}\frac{\partial p}{\partial Z}\right)+\frac{1}{{\left(R+kbZ-kb\right)}^{2}}\frac{\partial }{\partial \theta }\left(pQh^{3}\frac{\partial p}{\partial \theta }\right)=6\mu \omega \frac{\partial \left(ph\right)}{\partial \theta }+12\mu \frac{\partial \left(ph\right)}{\partial t}$$

where *μ* is the viscosity of the gas, *p* is the pressure, *h* is the film thickness, and *Q* is the flow rate coefficient for the Poiseuille flow, calculated by:

(5) $$Q=1+\frac{6.0972}{D}+\frac{2.40804}{D}\ln\left(1+\frac{1.2477}{D}\right),\;D=\frac{ph}{\mu \sqrt{2R_{g}T_{a}}}$$

where *D* is the inversed Knudsen number, with the gas constant *R*_g_ = 8.314 J/(mol·K).

The film thickness is expressed as follows:

(6) $$h=c+u\cdot n+h_{g}$$

where ***n*** is the normal unit vector of the bearing surface, which points to the rotor obtained by

(7) $$n=\left(\begin{array}{ccc} \cos\theta , & \sin\theta , & {\left(-1\right)}^{\xi }k \end{array}\right)/\sqrt{1+k^{2}}$$

where *ξ* is the number identifying the bearing, with *ξ =* 1 for the bearing in the positive *s*-axis and *ξ =* 2 for the bearing in the negative *s*-axis.

Then the bearing force and the bearing moment is calculated by

(8) $$F=\sum_{\xi =1}^{2}\iint_{\Omega }pndA$$

(9) $$M_{b}=\sum_{\xi =1}^{2}\iint_{\Omega }pn\times xdA$$

where Ω is the conical surface of the bearings, ***x*** = (*i*, *o*, *s*) is the position vector on the conical surface, obtained by the following relationship between O*θZ* and O_b_*ios:*

(10) $$\{\begin{array}{l} i=\left(R+k_{t}bZ-k_{t}b\right)\cos\theta \\ o=\left(R+k_{t}bZ-k_{t}b\right)\sin\theta \\ s=-{\left(-1\right)}^{\xi }\left(bZ+d/2\right) \end{array}$$

It could be obtained from Equations (7) and (10) that the vectors ***n***, ***x*** and *s*-axis are always in the same plane for any given point on the bearing surface. As a result, the *s*-component of ***M***_b_ is 0, and therefore the *s*-components of all the terms in Equation (3) are equal to 0 when the *s*-component of ***ω***_c_ is ignored.

Combining the Equations (2), (3), (8) and (9), the 5-DOF motion is governed by the following dynamic Equation

(11) $$m\frac{d^{2}q}{dt^{2}}+G\frac{dq}{dt}=Q_{b}+Q_{c}$$

where ***q*** = (*u_i_*, *u_o_*, *u_s_*, *φ_i_*, *φ_o_*) is state vector of 5-DOF motion, including translation *u_i_*, *u_o_*, *u_s_*, with the unit m, and tilting motion *φ_i_*, *φ_o_*, with the unit rad. ***m*** is the mass matrix, ***G*** is the gyroscopic matrix, ***Q***_b_ is the generalized bearing force vector, ***Q***_c_ is the generalized inertial force vector caused by the 5-DOF motion of the carrier. There is no control in this 5-DOF system. The matrixes and vectors are expressed as follows

(12) $$\begin{array}{l} \begin{array}{ccc} m=\left(\begin{array}{lllll} m & & & & \\ & m & & & \\ & & m & & \\ & & & J_{d} & \\ & & & & J_{d} \end{array}\right), & G=\left(\begin{array}{lllll} 0 & 0 & 0 & 0 & 0\\ 0 & 0 & 0 & 0 & 0\\ 0 & 0 & 0 & 0 & 0\\ 0 & 0 & 0 & 0 & J_{p}\omega \\ 0 & 0 & 0 & -J_{p}\omega & 0 \end{array}\right), & Q_{c}=\left(\begin{array}{l} -mf_{i}\\ -mf_{o}\\ -mf_{s}\\ J_{d}\frac{d\omega_{ci}}{dt}+J_{p}\omega \omega_{co}\\ J_{d}\frac{d\omega_{co}}{dt}-J_{p}\omega \omega_{ci} \end{array}\right), \end{array}\\ Q_{b}=\sum_{\xi =1}^{2}\iint_{\Omega }\frac{p}{\sqrt{1+k^{2}}}\left(\begin{array}{l} \cos\theta \\ \sin\theta \\ {\left(-1\right)}^{\xi }k\\ -{\left(-1\right)}^{\xi }(Rk_{t}+k_{t}^{2}bZ-k_{t}^{2}b+bZ+d/2)+\sin\theta \\ {\left(-1\right)}^{\xi }(Rk_{t}+k_{t}^{2}bZ-k_{t}^{2}b+bZ+d/2)\cos\theta \end{array}\right)dA \end{array}$$

### 3.2. Numerical Method

The rotor motion is influenced by the bearing force and the bearing moment, and vice versa. In addition, the coupling between translation and tilting motion is not negligible considering the cross stiffness and nonlinearity of gas-dynamic bearings. Therefore, Equations (4)–(6) and (12) need to be solved simultaneously. An explicit scheme with a tiny time step is adopted considering the rotor moves fast. The time step is set to 1 μs, scilicet 0.05% of a rotation period with rotating speed of rotor *n*_r_ = 30,000 r/min. The Reynolds Equation (4) is solved with the finite differential method in every time step to obtain the gas film pressure. The grid system with 180 × 31 nodes and the coordinate system O*θZ* for numerical calculation is shown in Figure 4. The boundary condition for Equation (4) is expressed as Equation (13), scilicet adopting periodic boundary in the circumferential direction and setting the pressures in both head faces equal to the ambient pressure.

(13) $$\left\{ \begin{array}{l} p(0,Z,t)=p(2\pi ,Z,t)\\ p(\theta ,0,t)=p(\theta ,1,t)=p_{a} \end{array}\right.$$

The initial condition is obtained by conducting a steady-state study with specific force ***f***_1_ and carrier angular velocity ***ω***_c1_. The following condition can be obtained for a steady state.

(14) $$\frac{\partial u}{\partial t}|{}_{t=0}=\frac{\partial^{2}u}{\partial t^{2}}|{}_{t=0}=\frac{\partial \varphi }{\partial t}|{}_{t=0}=\frac{\partial^{2}\varphi }{\partial t^{2}}|{}_{t=0}=0$$

By plugging Equation (14) into Equations (2) and (3), the initial values are obtained as bearing force ***F***(0) = *m**f***_1_ and ***M***_b_(0) = −*J*_p_***ω*** × ***ω***_c1_. The steady-state equilibrium position corresponding to ***F***(0) and ***M***_b_(0) is solved by the iteration method to initialize the displacement and tilting angle. The initial values of ***u*** and ***φ*** are guessed and compared with the corresponding bearing force and bearing moment until they are equal to ***F***(0) and ***M***_b_(0). The perturbation method is employed to estimate the stiffness matrix and damping matrix and improve the efficiency of the iteration. The initial gas film thickness and pressure are obtained by solving Equations (4) and (6) with the initial displacement and tilting angle.

**Figure 5:**
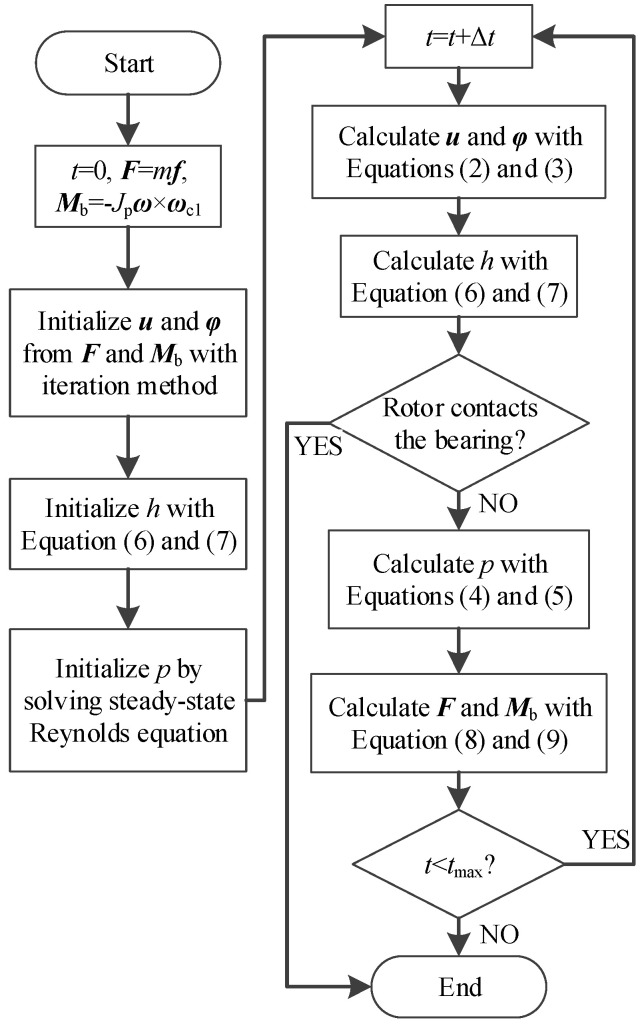
Calculation flow chart.

The steps to solve the govern Equations could be summed up based on all the aforementioned theories, as shown in Figure 5.

## 4. Results and Discussion

Based on the analytical theory, numerical procedures were programmed with MATLAB. The main parameter values adopted for the numerical simulation are listed in Table 1.

### 4.1. Hysteresis Loops of the Gas-Dynamic Bearings

**Figure 6:**
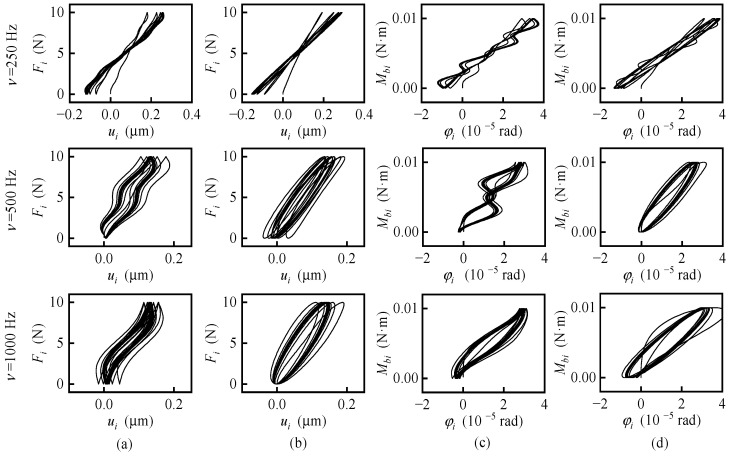
Hysteresis loops of the gas-dynamic bearings. (**a**) With a triangular perturbation of the force; (**b**) with a harmonic perturbation of the force; (**c**) with a triangular perturbation of the moment; (**d**) with a harmonic perturbation of the moment.

**Figure 7:**
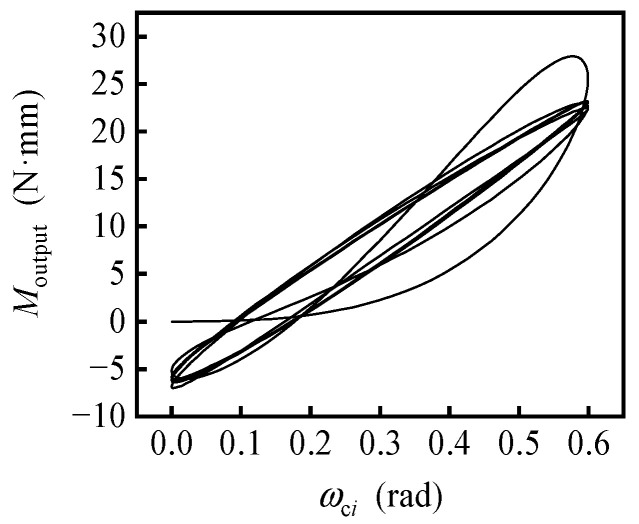
Hysteresis loops of the gyroscope.

The nonlinear and complex response characteristics of the gas-dynamic bearing gyroscope are largely caused and reflected by the hysteresis effect of the gas-dynamic bearings. Four kinds of one-directional perturbation were added, including triangular perturbation of the force *F_i_*(*t*) = *F*_max_tri(*νt*), harmonic perturbation of the force *F_i_*(*t*) = *F*_max_ − *F*_max_cos(2π*νt*), triangular perturbation of the moment *M*_b*i*_(*t*) = *M*_bmax_tri(*νt*) and harmonic perturbation of the moment *M*_b*i*_(*t*) = *M*_bmax_ − *M*_bmax_cos(2π*νt*). The function tri(*) is defined by tri(*x*) = 2{*x*} for 0 ≤ {*x*} < 0.5 and tri(*x*) = 2 − 2{*x*} for 0.5 ≤ {*x*} < 1, where {*} is the decimal part function. The hysteresis loops are presented in Figure 6, calculated with the maximum of the force *F*_max =_ 10 N, the maximum of the moment *M*_bmax =_ 0.01 N·m and the frequency *ν =* 250 Hz, 500 Hz, 1000 Hz, respectively. In general, the higher the frequency of excitation is, the bigger the area of the hysteresis loop is, which is consistent with the conclusion of reference [31]. The amplitude of rotor vibration is larger with the frequency *ν =* 250 Hz, the half of the rotating speed of the rotor, which could cause the superposition of the force vibration and half-frequency whirl. The hysteresis curves of triangular perturbation in Figure 6a,c show more tortuous and nonlinear compared with the curves of harmonic perturbation in Figure 6b,d. As the moment *M*_output_ to determine the output current is expressed by *M*_output =_ −*M_o_* − ***u*** × ***F***·***e***, the hysteresis effect of the gas-dynamic bearing causes the hysteresis effect of the gyroscope. For harmonic perturbation with *ν* = 1000 Hz, the corresponding hysteresis loop of the gyroscope is shown in Figure 7, and it has a similar trend to Figure 6d. As a result, the output of the gyroscope is not only related to the current motion state but also to the previous motion state. Therefore, it is necessary to study the transient nonlinear behavior of gas-dynamic bearing gyroscope.

### 4.2. Response with Uniformly Changed Specific Force

It is assumed that in time *t* < 0, a steady state of the rotor motion in the reference frame O_b_*ios* is obtained with a constant specific force ***f***_1_ and a constant angular velocity ***ω***_c1_. In time 0 ≤ *t* < *t*_1_, the specific force and the angular velocity change with a constant rate. Until *t = t*_1_, the specific force is changed to ***f***_2_, the angular velocity is changed to ***ω***_c1_, and they maintained these values for *t* > *t*_1_. Results in Section 4.2, Section 4.3 and Section 4.4 are obtained based on the assumption, the trajectory and the phase portrait of the rotor center are obtained, and the curves of the net force and the interference torque are plotted.

**Figure 8:**
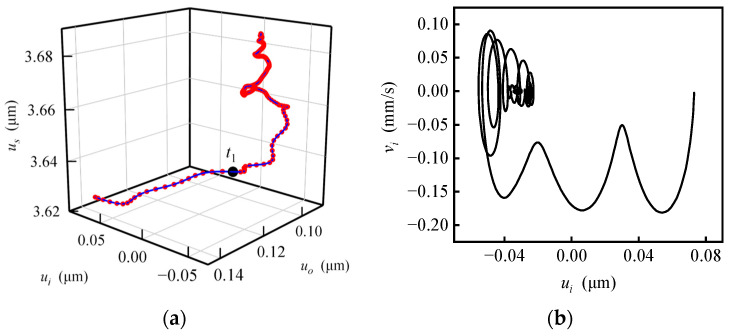

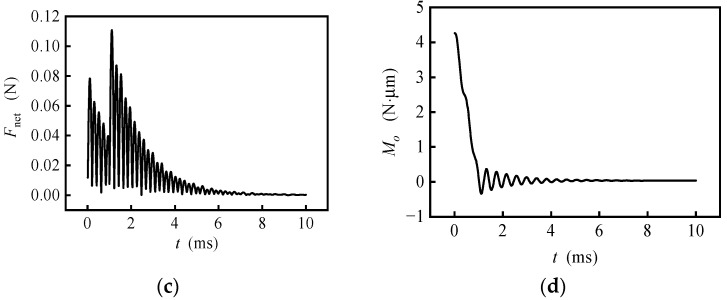
Response with uniformly changed specific force. (**a**) Trajectory of the rotor center; (**b**) phase portrait of the rotor center; (**c**) time response of the net force; (**d**) time response of the interference torque.

Responses with uniformly changing specific force are shown in Figure 8, calculated with ***f***_1_ = (−20 −20 −20) m/s^2^, ***f***_2_ = (0 −20 −20) m/s^2^, *t*_1_ = 1 ms and ***ω***_c1_ = ***ω***_c2_ = **0**. When the specific force changes (before *t*_1_), the rotor moves smoothly to the new balance position, but mainly in the radial direction, while the axial displacement is small. When the specific force stops to change (after *t*_1_), the rotor whirls and spirals up along the axial direction and finally stops in the new equilibrium position. In Figure 8b, when the specific force changes, the speed of the rotor along the *i*-axis still fluctuates to a certain extent, which is always negative; that is, the rotor moves towards the balance position with speed fluctuating as a result of natural vibration. When the specific force stops changing, the phase trajectory of the rotor oscillates in a small range and tends to converge. In Figure 8c, the net force fluctuates obviously, which shows a coincidence with the speed fluctuation in Figure 8b. The fluctuation decreases in intervals (0, *t*_1_) and (*t*_1_, ∞) and instantaneously increases at *t*_1_. In Figure 8d, the change of the specific force is nearly linear with time, while the specific force changes and fluctuates in a similar manner with the linear underdamping vibration when the specific force stops changing. The method to identify the linear underdamping vibration law is introduced in a previous study [8].

**Figure 9:**
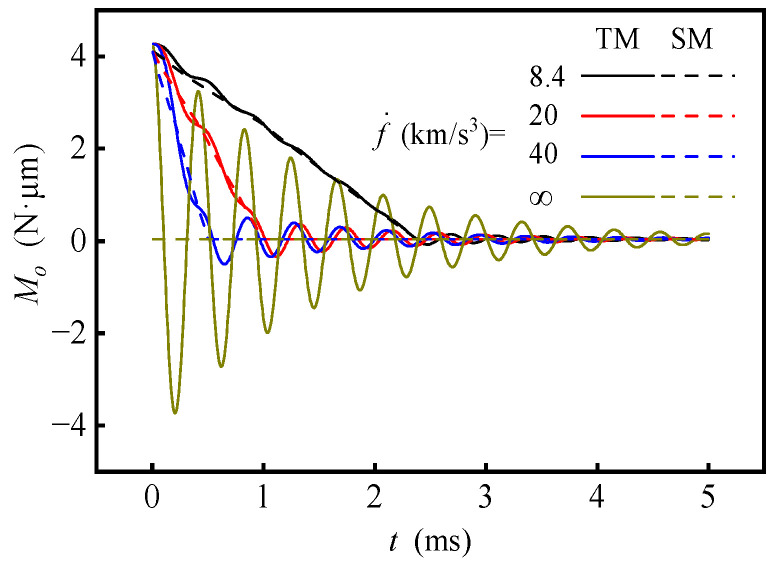
Interference torque under different change rates of specific force.

The interference torque curves under different change rate of specific force are shown in Figure 9, calculated with fixed ***f***_1_ = (−20 −20 −20) m/s^2^ and ***f***_2_ = (0 −20 −20) m/s^2^ and varying *t*_1_. The change rate of specific force is expressed by $\dot{f}$ = |***f***_1_ − ***f***_2_|/*t*_1_, and $\dot{f}$ = ∞ indicates the sudden change of specific force. The results are obtained by the following two methods: (1) the aforementioned transient method (TM) solving the transient Reynolds equation and rotor dynamic equation simultaneously, (2) the steady-state method (SM) ignoring the squeezing effect of the gas film proposed in a previous study [7]. The fluctuation of the interference torque curve increases with the increase of the specific force change rate. The sudden change of specific force causes larger fluctuation than the others, while the uniform change of specific force will not cause severe vibration. Even if the specific force change rate is as high as 40 km/s^3^, it should not be simplified as a sudden change. The interference torque curve is approximately a straight line when the specific force changes uniformly and remains constant when the specific force stops to change. The interference torque curves obtained by the two methods almost coincide with $\dot{f}$ < 8.4 km/s^3^, which is consistent with the deduction in reference [7]. 

### 4.3. Response with Uniformly Changed Angular Velocity

**Figure 10:**
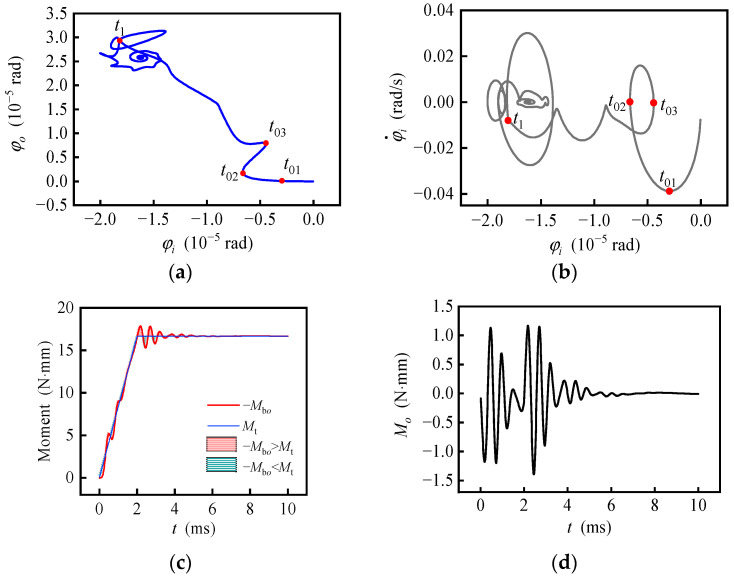
Response with uniformly changed angular velocity. (**a**) Tilting motion track; (**b**) phase portrait of tilting motion; (**c**) the reaction moment of the bearing moment and its theoretical value; (**d**) time response of the interference torque.

Responses with uniformly changing carrier angular velocity around *i*-axis and fixed specific force are shown in Figure 10, calculated with ***f***_1_ = ***f***_2_ = **0**, *t*_1_ = 2 ms, ***ω***_c1_ = (0 0 0) rad/s and ***ω***_c2_ = (1 0 0) rad/s. When angular velocity begins to increase in the positive direction around *i*-axis, the rotor rotates in the negative direction around the *i*-axis relative to the carrier due to inertia. The relative angular velocity increases with the increase of the carrier angular velocity, for the period of time 0~*t*_01_ shown in Figure 10a,b). The angular velocity of the rotor around the *i*-axis relative to the inertial reference frame causes the gyroscopic moment around the *o*-axis, which makes the rotor start to move around the *o*-axis for the period of time *t*_01_~*t*_02_. When the angular velocity of the rotor exceeds the angular velocity of the carrier because of the elasticity of the gas film, the gyro moment around the *o*-axis increases, and then the angular velocity of the rotor around the *o*-axis also increases, resulting in the gyroscopic moment around negative *i*-axis. Therefore, the angular velocity of the rotor relative to the carrier around the *i*-axis quickly returns to a negative value for *t*_02_~*t*_03_. The rotor repeatedly adjusts and approaches a dynamic balance state gradually. In this state, the relative angular displacement of the rotor is always ahead of the quasi-equilibrium position under the angular velocity of the carrier so as to provide the acceleration relative to the inertial reference frame, while the constantly changing bearing torque around the *o*-axis keeps balance with the gyro moment, for *t*_03_~*t*_1_. When the angular velocity of the carrier stops changing, the angular velocity of the rotor continues to increase around the *i*-axis under the action of the bearing moment, but the decrease of the bearing moment makes the angular acceleration decrease rapidly. After repeated adjustment, the rotor trajectory finally converges to the quasi-equilibrium position corresponding to ***ω***_c2_. Therefore, when the angular velocity of the carrier changes, the angular motion of the rotor is influenced by unequal elasticity, whirl and gyroscopic precession, and the angular motion trajectory of the rotor is more complex. In the inertial reference frame, the gyro precession and half-frequency whirl directions caused by small perturbation are both consistent with the rotation direction. However, for the gyroscope with a non-inertial reference frame fixed on the carrier, the direction of gyro precession and half-frequency whirl may be inconsistent. For example, in the period of time *t*_01_~*t*_02_, the precession of the gyroscope makes the rotor rotate positively around the *o*-axis while the whirl makes the rotor rotate negatively around the *o*-axis.

Without the influence of specific force, only the reaction moment of the bearing moment acts directly on the gyro unit and produces the feedback control current to estimate the carrier angular velocity, which is plotted to be compared with the theoretical value *M*_t_ = *H*_r_*ω*_c*i*_ in Figure 10c. The curve of –*M*_b*o*_ fluctuates around the curve of *M*_t_, and they are merged together a few microseconds after the angular velocity stops changing, which also indicates that the output of the gyroscope is influenced by the previous motion state. The interference torque curve is shown in Figure 10d. The reaction moment of bearing moment fluctuates around the theoretical value. The amplitude of the interference torque increases suddenly when the carrier begins to rotate and stops accelerating but decreases gradually in the period when the angular velocity changes uniformly or no longer.

**Figure 11:**
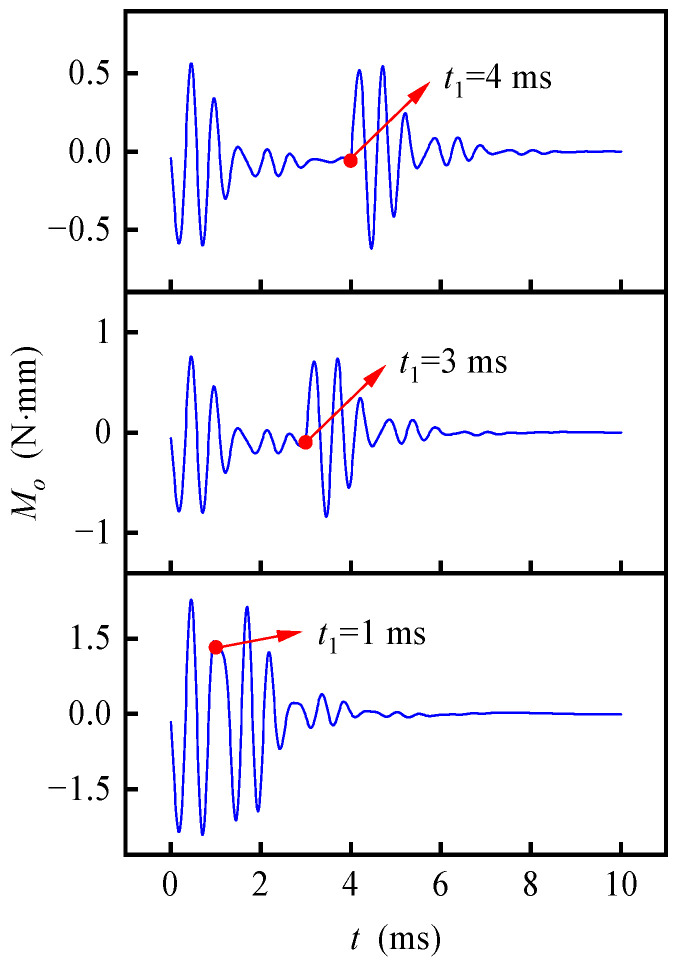
Rotor angular trajectory and interference torque under different angular acceleration.

Figure 11 shows the interference torque curves influenced by a variety of angular acceleration, obtained with ***f***_1_ = ***f***_2_ = **0**, ***ω***_c1_ = (0 0 0) rad/s, ***ω***_c2 =_ (1 0 0) rad/s and *t*_1_ = 1, 3, 4 ms, i.e., $\dot{\omega }$_c_ = 1000, 333.3, 250 rad/s^2^, which indicates that a smaller angular acceleration causes a smaller fluctuation of interference torque.

**Figure 12:**
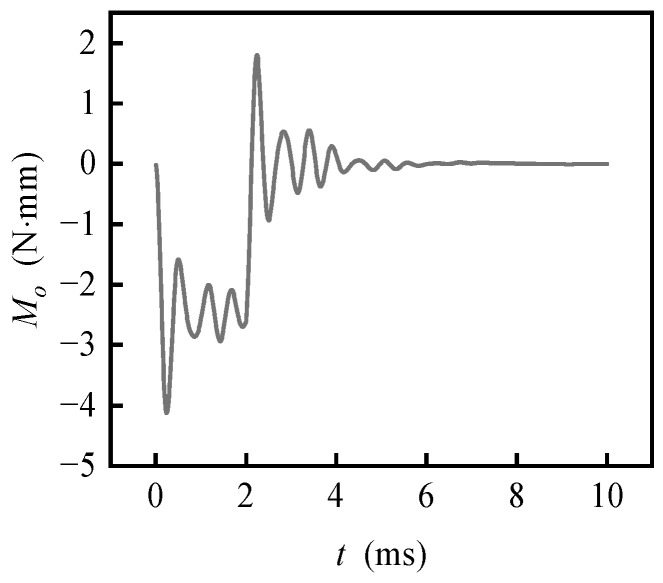
Transient interference torque with carrier angular velocity uniformly changing along *o*-axis.

Figure 12 shows the interference torque curves caused by an *o*-axis change of carrier angular velocity, obtained with ***f***_1_ = ***f***_2_ = **0**, ***ω***_c1_ = **0**, ***ω***_c2_ = (0 1 0) rad/s and *t*_1_ = 2 ms. The Δ***ω***_c_ around the *o*-axis will produce a negative interference torque around the *o*-axis, which is caused by the inertia moment of the rotor, estimated by −*J*_d_ $\dot{\omega }$_c*o*_ = −2.25 N·mm. 

### 4.4. Response with Uniformly Changed Specific Force and Angular Velocity

**Figure 13:**
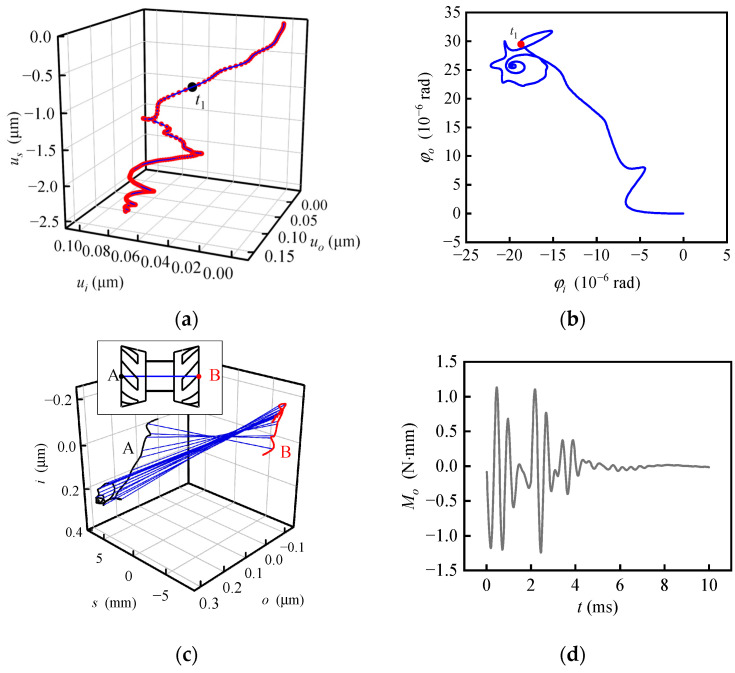
Response with specific force and carrier angular velocity changing simultaneously and uniformly. (**a**) Trajectory of the rotor center; (**b**) tilting motion track; (**c**) motion of the rotor centerline; (**d**) time response of the interference torque.

If the specific force and angular velocity of the carrier change uniformly at the same time, response is presented in Figure 13, calculated with ***f***_1_ = **0**, ***f***_2_ = (0 0 10) m/s^2^, *t*_1_ = 2 ms, ***ω***_c1_ = **0** and ***ω***_c2_ = (1 0 0) rad/s. The results show that the trajectory of the rotor has a similar trend with the case of only specific force change, while the rotor angular motion track has a similar trend with the case of only the carrier angular velocity change. However, the characteristics of coupling between the two factors are still obvious. For example, after *t*_1_ in Figure 13b, the center of the track circle caused by half-frequency whirl shifts to one side with time. Because of the coupling effect of translation and tilt, part of the gas film becomes very thin, resulting in significantly nonlinear elasticity. The larger the radius of the whirl track is, the greater the difference of stiffness for each point on the trajectory circle is, and the greater the offset of the trajectory circle is, which causes the center of the trajectory circle to shift with time. With the decrease of whirl radius, the center of the trajectory circle gradually returns to the quasi-equilibrium position.

## 5. Conclusions

(1) The gas-dynamic bearings show a significant hysteresis effect with a perturbation of bearing force or bearing moment, which results in the hysteresis effect of the gyroscope. As a result, the output of the gyroscope is not only related to the current motion state but also to the previous motion state.

(2) The change of the specific force is nearly linear with time, while the specific force changes and fluctuates in a similar manner with the linear underdamping vibration when the specific force stops changing. Even if the specific force change rate is up to 40 km/s^3^, it should not be simplified as a sudden change. The interference torque curves obtained by TM and SM almost coincide when the specific force change rate is less than 8.4 km/s^3^, which is consistent with the applicable domain of SM deducted in the previous study.

(3) The amplitude of the interference torque increases suddenly when the carrier begins to rotate and stops accelerating but decreases gradually in the period when the angular velocity changes uniformly or no longer. An *o*-axis change of carrier angular velocity will produce a negative interference torque.

For engineering practice, the linear and quadratic error should be tested by experiment first. Then, the method in this paper could be used to explore the trend of the nonlinear error caused by the flexibility of the gas-dynamic bearing. Moreover, finally, an experiment is needed to verify the numerical results. The advantage of using this method rather than only experiments is to accurately reveal the complex trend of the nonlinear error with fewer experiments.

## Figures and Tables

**Table 1:** Parameters of the rotor-bearing system.

Parameter	Value
**Bearing**	
Bottom radius, *R* (mm)	7.5
Bearing taper, *k*	0.25
Bearing width, *b* (mm)	6
Bearing clearance, *c* (μm)	2
Spacing between the bearings, *d* (mm)	8
Groove depth, *h*_g_ (μm)	1
Number of grooves on each bearing, *N*_g_	6
Groove angle, *β*_g_ (°)	45
**Rotor**	
Mass, *m* (g)	60
Rotating speed, *n*_r_ (r/min)	30,000
Moment of inertia around *i*-axis or *o*-axis, *J*_d_ (kg·m^2^)	4.4533 × 10^−6^
Moment of inertia around *s*-axis, *J*_p_ (kg·m^2^)	5.308 × 10^−6^
**Lubricants**	
Viscosity, *μ* [Pa⋅s]	1.79 × 10^−5^
Ambient pressure, *P*_a_ [Pa]	1.013 × 10^5^
